# Elimination of Ribosome Inactivating Factors Improves the Efficiency of *Bacillus subtilis* and *Saccharomyces cerevisiae* Cell-Free Translation Systems

**DOI:** 10.3389/fmicb.2018.03041

**Published:** 2018-12-18

**Authors:** Tetiana Brodiazhenko, Marcus J. O. Johansson, Hiraku Takada, Tracy Nissan, Vasili Hauryliuk, Victoriia Murina

**Affiliations:** ^1^Department of Molecular Biology, Umeå University, Umeå, Sweden; ^2^Laboratory for Molecular Infection Medicine Sweden (MIMS), Umeå University, Umeå, Sweden; ^3^Institute of Technology, University of Tartu, Tartu, Estonia; ^4^Department of Molecular Biosciences, The Wenner-Gren Institute, Stockholm University, Stockholm, Sweden; ^5^School of Life Sciences, University of Sussex, Brighton, United Kingdom

**Keywords:** HPF, Stm1, *Bacillus subtilis*, *Saccharomyces cerevisiae*, cell-free translation system

## Abstract

Cell-free translation systems based on cellular lysates optimized for *in vitro* protein synthesis have multiple applications both in basic and applied science, ranging from studies of translational regulation to cell-free production of proteins and ribosome-nascent chain complexes. In order to achieve both high activity and reproducibility in a translation system, it is essential that the ribosomes in the cellular lysate are enzymatically active. Here we demonstrate that genomic disruption of genes encoding ribosome inactivating factors – HPF in *Bacillus subtilis* and Stm1 in *Saccharomyces cerevisiae* – robustly improve the activities of bacterial and yeast translation systems. Importantly, the elimination of *B. subtilis* HPF results in a complete loss of 100S ribosomes, which otherwise interfere with disome-based approaches for preparation of stalled ribosomal complexes for cryo-electron microscopy studies.

## Introduction

Cell-free translation systems based on cellular lysates optimized for *in vitro* protein synthesis have multiple applications both in basic and applied science, ranging from studies of translational regulation (Wu et al., 2007) to cell-free production of recombinant proteins (Pedersen et al., 2011) and ribosome-nascent chain complexes (Rutkowska et al., 2009). The preparation of cell-free translation systems is a compromise between, on one hand, the desired properties, such as high synthetic activity and reproducibility of the system and, on the other hand, simplicity of generating robust extracts as well as economic considerations. In laboratory settings, the most convenient and readily accessible method of producing biomass is by growing cells in a batch format in flasks. In this case, large-scale production of highly translationally active exponentially growing cells can be challenging due to relatively low yields. To maximize extract yields, one can harvest cultures in late exponential/early stationary phase. While this provides more biomass, there is a drawback in that cells often reduce their translational capacity during slow growth (Dai et al., 2016). Importantly, the translational activity decreases upon exiting rapid exponential growth – and an important mechanism at play is the reduction of the concentration of active ribosomes via ribosomal sequestration into inactive complexes by dedicated regulatory protein factors.

Bacteria reduce their translational capacity either by directly inactivating 70S ribosomes (Agafonov et al., 1999) or forming inactive ribosome dimers, so-called 100S ribosomes (Ueta et al., 2010; Gohara and Yap, 2018). In *Escherichia coli*, formation of inactive 70S is mediated by protein Y (YfiA) that in response to cold shock associates with vacant ribosomes and precludes tRNA and mRNA binding (Vila-Sanjurjo et al., 2004). 100S ribosome formation in Gammaproteobacteria is mediated by two cooperating factors: the Hibernation Promoting Factor (HPF; homologous to YfiA (Ueta et al., 2008)) and the Ribosome Modulation Factor (RMF) (Wada et al., 1990). In *E. coli* the 100S is highly unstable, and therefore formation of the 100S in the stationary phase does not significantly reduce the efficiency of translational lysates (Failmezger et al., 2017). In the majority of bacterial species, 100S formation is mediated by one factor – HPF – the long version of the “short” HPF present in the Gammaproteobacteria (Akanuma et al., 2016; Beckert et al., 2017; Khusainov et al., 2017). Neither the *Staphylococcus aureus* nor the *Bacillus subtilis* genomes encode YfiA. *S. aureus* and *B. subtilis* 100S are considerably more stable, therefore 100S formation can potentially have a significant impact on translation efficiency in lysates (Ueta et al., 2010; Akanuma et al., 2016; Basu and Yap, 2016).

In budding yeast, the Stm1 protein acts as a translational repressor (Balagopal and Parker, 2011) and is recruited to 80S ribosomes upon nutrient limitation (Ben-Shem et al., 2011; van den Elzen et al., 2014). Rather than causing dimerization, Stm1 and its metazoan ortholog SERBP1 occlude the mRNA-binding channel in both the A- and P-site sites thus forming stable inactive 80S particles (Ben-Shem et al., 2011; Anger et al., 2013). As expected for a ribosome inactivation factor, when Stm1 is added to yeast translational extracts, their activity is strongly inhibited (Balagopal and Parker, 2011).

We reasoned that disrupting the genes encoding for ribosome inactivating factors would yield more reproducible and active bacterial (*B. subtilis* Δ*hpf*) and yeast (*Saccharomyces cerevisiae stm1*Δ) cell-free translation systems. Addition of either HPF (Ueta et al., 2010) or Stm1 (Balagopal and Parker, 2011) purified proteins to cell-free translation systems *in trans* is known to inhibit the efficiency of protein production. The use of a Δ*hpf* strain could be an especially promising strategy for improving the translation efficiency of *B. subtilis* lysates. Since this bacterium already expresses its HPF during exponential growth (although at significantly lower levels than in stationary phase) (Akanuma et al., 2016), it is expected that cell-free translation systems prepared from the Δ*hpf* strain would be more active than those prepared from the wild type strain regardless of growth phase.

Another motivation for examining translational efficiency of lysates prepared from the Δ*hpf B. subtilis* strain is the potential utility of Δ*hpf* lysates for generating stalled ribosomal complexes. A popular strategy for preparation of stalled ribosomal complexes utilizes cell-free translation of dicistronic *2Xerm*-mRNA encoding two identical Erm-stalling leader peptides (Arenz et al., 2014; Crowe-McAuliffe et al., 2018). Stalled ribosomal dimers formed in the presence of the antibiotic erythromycin are readily separated from 70S monosomes – but not from 100S particles – on sucrose gradients. Use of the Δ*hpf* strain lacking 100S ribosomes to generate extracts avoids this problem.

## Results

### Elimination of HPF Improves the Efficiency of *B. subtilis* Coupled Transcription-Translation System

**FIGURE 1:**
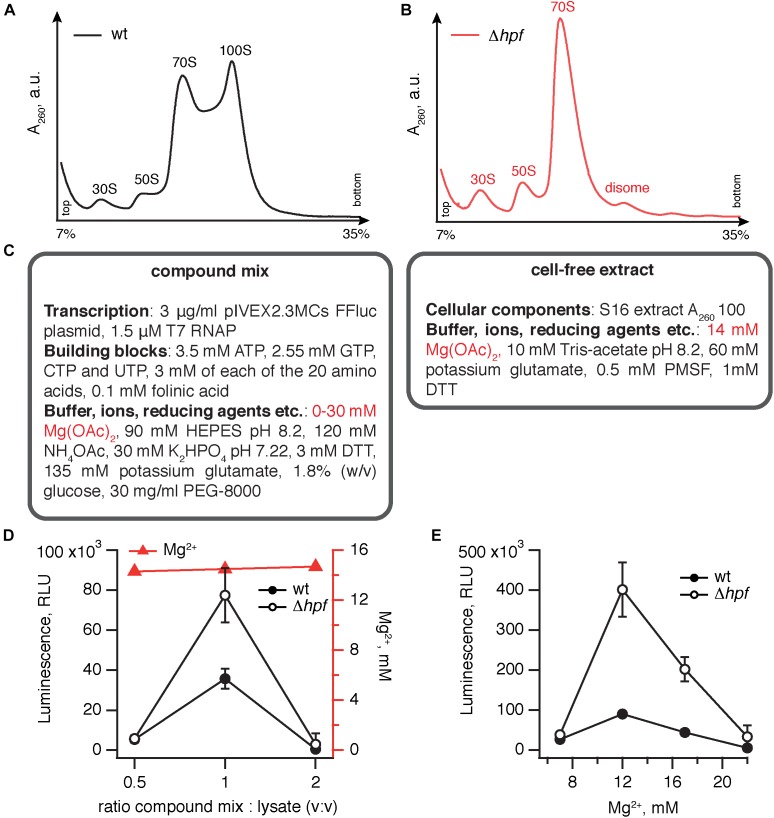
Elimination of HPF improves the efficiency of *B. subtilis* coupled transcription-translation system. Polysome profile analysis of translational lysates prepared from wild type **(A)** and Δ*hpf* **(B)** lysates demonstrates strictly HPF-dependent 100S ribosomes formation. **(C)** A cell-free translation system was assembled by combining the compound mix with the cell-free extract. The efficiency of translation was quantified by the activity of the firefly luciferase using the Steady-Glo Luciferase Assay (Promega). Titrations of the compound mix to cell-free extract ratio **(D)** and magnesium ion concentration **(E)** in the cell-free translation system. Luminescence readings were taken after incubation for 1 h at 37°C. Error bars indicate the standard error of the geometric mean of biological replicates, i.e., independently prepared cell-free extracts (*n* ≥ 3).

We opted for a coupled transcription-translation system utilizing the pIVEX2.3MCs FFluc plasmid (Starosta et al., 2010) that encodes the firefly luciferase ORF preceded by *B. subtilis* optimized ribosome binding site (RBS). Transcription of the mRNA is driven by recombinant T7 RNA polymerase added to the lysate (Antoun et al., 2004) and the efficiency of protein synthesis was quantified by measuring the luminescence of the translated luciferase protein. For preparing cell-free extracts we used the wild type 168 *B. subtilis* strain and an isogenic Δ*hpf* mutant that displays no growth defect, except for a moderate increase in the lag phase (Akanuma et al., 2016). The cells were collected at OD_600_ 1.8–2.2 and the lysates prepared as described in the Materials and Methods section. The polysome profile analyses show that in the case of the wild type strain, the 100S peak is dominant and stable (Figure 1A), while in the Δ*hpf* mutant it is lacking altogether (Figure 1B). Importantly, the situation is dramatically different in the case of *E. coli*: when lysates are prepared from the stationary phase *E. coli* cells, no 100S peak is observed (Failmezger et al., 2017).

Our protocol for preparation of a *B. subtilis* transcription-translation system was based on that of Krinsky et al. (2016), which is specifically optimized to be rapid, cheap and relatively efficient – despite lacking a dedicated ATP regeneration system, it was able to generate 40–150 μg-protein/mL. The system is a binary mixture of cell-free extract and compound mix (Figure 1C). The cell-free extract contains a full set of cellular components carrying out protein synthesis, i.e., ribosomes, tRNAs, aminoacyl tRNA synthetases, methionyl-tRNA formyltransferase and translational factors. The compound mix contains (i) inorganic ions, importantly Mg^2+^, the key player in ribosomal function (Nierhaus, 2014) (ii) buffering (HEPES) and reducing (DTT) agents (iii) NTPs that serve both as the energy source and as the building blocks for mRNA synthesis (iv) template DNA in a form of pIVEX2.3MCs FFluc plasmid supplemented with recombinant T7 RNAP polymerase (v) amino acids and folinic acid that serve as building blocks for protein synthesis and, finally, (vi) stabilizing agents such as PEG-8000 and glucose.

To test the robustness of the effect of HPF loss on the efficiency transcription-translation system, we performed two titrations of the key components. As a first step, we have varied the ratio between the compound mix and cell-free extract (Figure 1D). To ensure the robustness of our results, we used at least three independently prepared cell-free extracts for each titration point. At a 1:1 ratio the activity is optimal, and the Δ*hpf* lysate is robustly approximately twofold more active.

The second titration step was aimed at identifying the optimal concentration of magnesium ions (Zaher and Green, 2014). Magnesium is important both for optimizing transcriptional and translational efficiency in the coupled system. While using *in vitro* transcribed mRNA it is possible to discriminate between Mg^2+^ effects on transcription and translation, the specific effect of HPF elimination is unlikely to act on the transcriptional level.

Maintaining the ratio between the extract and compound mix at 1:1, we titrated the final concentration of Mg^2+^ from 7 to 22 mM (Figure 1E). While the activity of the Δ*hpf* lysate peaks at 12 mM Mg^2+^, reaching an excess of 400,000 relative light units (RLU; the term “relative” is used since rather than providing absolute quantification of photons, the efficiency of detection is specific for a particular luminometer instrument), the Δ*hpf* lysate is more active than the wild type at all magnesium concentrations tested. Therefore, we concluded that loss of HPF robustly improves the efficiency of *B. subtilis* lysates prepared from stationary phase cells.

### Elimination of Stm1 Improves the Efficiency of *S. cerevisiae* Translation System

**FIGURE 2:**
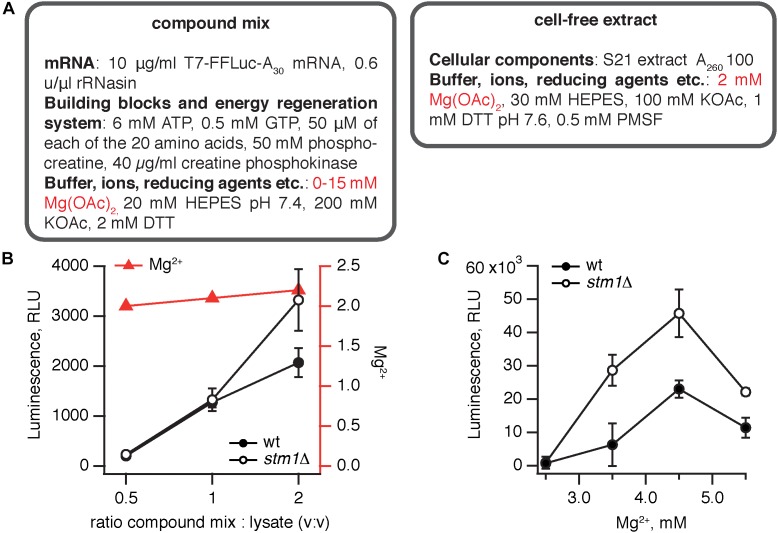
Elimination of Stm1 improves the efficiency of *S. cerevisiae* translation system. **(A)** A cell-free translation system was assembled by combining the compound mix with the cell-free extract. The efficiency of translation was quantified by the activity of firefly luciferase using the Steady-Glo Luciferase Assay (Promega). Titrations of the compound mix to cell-free extract ratio **(B)** and magnesium ion concentration **(C)** in the cell-free translation system. Luminescence readings were taken after incubation for 1 h at 25°C. Error bars indicate the standard error of the geometric mean of biological replicates, i.e., independently prepared cell-free extracts (*n* ≥ 3).

The *S. cerevisiae stm1*Δ strain was constructed by deleting the *STM1* gene in the wild type MBS (Iizuka and Sarnow, 1997) strain. We have opted for a translation system supplemented with an *in vitro* transcribed, capped polyadenylated luciferase mRNA. Capping mRNA dramatically increases the efficiency of translation (Tarun and Sachs, 1995) but cannot be performed *in situ* in the lysate and must be added enzymatically to the mRNA after transcription. The translation protocol was based on that of Wu et al. (2007). Just as the bacterial coupled translation-transcription system, the yeast translation system is also a binary mixture of cell-free extract and a compound mix (Figure 2A). Since transcription is performed separately, the compound mix contains only the two NTP species necessary for translation, i.e., GTP and ATP, and mRNA is stabilized by RNase inhibitor (rRNasin). Creatine phosphokinase and phosphocreatine serve an energy regeneration system.

Similarly to the *B. subtilis* system, we have performed two titrations, using at least three independently prepared cell-free extracts for each titration point: altering the ratio between the compound mix and cellular extract (Figure 2B) and the amount of Mg^2+^ in the extract (Figure 2C). The efficiency of the system gradually increases with the percentage of the compound mix in the reaction from 33 to 66% (Figure 2B). Notably, both at 33–50% compound mix there is no significant difference between the wild type and *stm1*Δ lysates; only in the presence of 66% compound mix does the *stm1*Δ translation reaction display higher activity. However, even in this case, the activity is two orders of magnitude lower than that seen in the *B. subtilis* system. A likely culprit is a sub-optimal concentration of Mg^2+^ ions; as the fraction of the compound mix increases, the Mg^2+^ concentration is moderately increased from 2 to 2.2 mM, following the concomitant increase in activity (Figure 2B). Therefore, we titrated Mg^2+^ from 2.5 to 5.5 mM (Figure 2C). The activity peaked at 4.5 mM Mg^2+^, reaching an excess of 40,000 RLU with the *stm1*Δ *S. cerevisiae* lysate robustly out-performing the wild type lysate.

## Discussion

Here we demonstrated that genomic disruption of genes encoding ribosome inactivating factors – HPF in *B. subtilis* and Stm1 in *S. cerevisiae* – moderately but robustly (*n* ≥ 3 biological replicates – i.e., independently prepared cell-free extracts – were used for each data point) improves the activity of bacterial and yeast translation systems. Both of the proteins directly inactivate the ribosomes and are to cell-free translation lysates when added *in trans* (Ueta et al., 2010; Balagopal and Parker, 2011). Importantly, elimination of HPF leads to a complete loss of 100S ribosomes, thus despite only a moderate increase in the overall translational efficiency, the Δ*hpf* lysate is particularly well-suited for disome-based approaches for preparation of stalled complexes for cryo-EM structural studies (Arenz et al., 2014; Crowe-McAuliffe et al., 2018).

The increase in efficiency of the delta Δ*hpf B. subtilis* system is modest compared to the effects of reducing cellular proteolytic or RNase activities: a well-proven approach for genetic manipulation of strains for *in vitro* translation, as exemplified by the *B. subtilis* WB800N strain lacking eight protease-encoding genes (Wu et al., 2002) that is used for cell-free translation (Kelwick et al., 2016) and the *E. coli* MRE600 strain with low RNase I activity (Kurylo et al., 2016) that is used for preparation of active ribosomes (Rivera et al., 2015) and translation systems (Castro-Roa and Zenkin, 2015). In the case of *B. subtilis* WB800N, the cell-free translation system prepared from this strain is 72 times more active than that made from the wild type (Kelwick et al., 2016) – a significantly larger improvement compared to the twofold effect observed upon *hpf* deletion. A possible next step in developing strains for more efficient cell-free translation system is by combining genetic modifications targeting proteolysis, RNA degradation and ribosome hibernation. In the case of the *S. cerevisiae* system, optimization strategies used in recent years have been focused on cultivation conditions, preparation of lysates and approaches for replenishment of substrates (Zemella et al., 2015). Therefore, even the moderate improvement in efficiency yielded by Stm1 elimination has significance for biotechnological applications.

## Materials and Methods

### Preparation 6His-Tagged Recombinant T7 RNA Polymerase (T7 RNAP)

*E. coli* BL21 strain (NEB) lacking the λDE3 lysogen was transformed with pQE30-T7RNAP plasmid (Amp) (Antoun et al., 2004). An overnight culture in LB media supplemented with 50 μg/mL of ampicillin was used to inoculate a large-scale culture in the same media grown at 37°C with shaking. At OD_600_ of 0.5–0.6 expression of 6His-tagged T7 RNAP was induced by addition of IPTG to a final concentration of 1 mM. After an additional 2 h growth, cells were harvested by centrifugation, resuspended in buffer A (150 mM NaCl, 100 mM Tris:HCl pH 7.5, 2 mM MgCl_2_, 1 mM ß-mercaptoethanol) supplemented with 0.1 mM PMSF, 35 μg/mL lysozyme and 1 u/mL DNase I and lysed by one pass via Stansted Fluid Power SFPH-10 Stansted Pressure Cell/Homogenizer (1.5 bar). After removal of the cell debris by centrifugation (35,000 rpm for 40′), the clarified lysate was loaded onto a 1 mL HisTRAP HP column (GE Healthcare) equilibrated in buffer A. The column was washed with high salt buffer B (same as buffer A except for the addition of 2 M NaCl), and the protein was eluted by a gradient of buffer C (buffer A supplemented with 0.5 M imidazole), pure fractions were pooled, concentrated and buffer-exchanged into storage buffer (100 mM KCl, 20 mM Tris:HCl pH 7.5, 5 mM MgCl_2_, 1 mM DTT, 50% glycerol) using 50 MWCO centricons (Amicon). The purity of protein preparations was assessed by SDS PAGE and spectrophotometrically (A_280/260_ ratio of approximately 1.8). The protein was stored at −20°C.

### Preparation of Firefly Luciferase mRNA for Use in Yeast Translational Lysates

Firefly luciferase mRNA containing a 30 nucleotide poly(A) tail was *in vitro* transcribed from the luciferase T7 control plasmid (Promega) linearized by Afel as a DNA template for the T7 RNA Polymerase (HiScribe^TM^ T7 High Yield) RNA Synthesis Kit. A typical 20 μL reaction containing 1 pmole DNA was incubated for 2 h at 37°C prior to mRNA isolation with MEGAclear^TM^ Kit (Ambion), followed by capping by the Vaccinia Capping System (NEB) and re-purification of mRNA with MEGAclear^TM^ Kit (Ambion). The quality of the final product was confirmed by denaturing agarose electrophoresis.

### *B. subtilis* and *S. cerevisiae* Strains

The wild type 168 (*trpC2*) *B. subtilis* strain was provided by Yuzuru Tozawa and the isogenic Δ*hpf* knockout *B. subtilis* strain RIK2508 (*trpC2* Δ*hpf*)) was provided by Fujio Kawamura (Akanuma et al., 2016).

A *S. cerevisiae* strain deleted for *STM1* (MJY1079, *MATa ura3-1 leu2-3,112 his3-11,15 trp1-1 ade2-1 can1-100 stm1::HIS3MX6* L-o M-o) was constructed by transforming the MBS strain (Iizuka and Sarnow, 1997) with a *stm1::HIS3MX6* DNA fragment PCR amplified from pFA6a-*HIS3MX6* (Wach et al., 1997). The oligonucleotides used were: 5′-AGTAGAAATAAACCAAGAAAGCATACACATTTTATTCTCACGGATCCCCGGGTTAATTAA-3′ and 5′TTATTGGATTCTTTCAGTTGGAATTATTCATATATAAGGCGAATTCGAGCTCGTTTAAAC-3′. The deletion was confirmed by PCR using primers that annealed outside of sequences present in the transformed DNA fragment.

### Preparation of *B. subtilis* Bacterial Cell-Free Extract

The lysate preparation protocol is based on that of Krinsky et al. (2016) with minor modifications. Fifty milliliter LB cultures of *B. subtilis* 168 wild type and Δ*hpf* strains were inoculated with single colonies from fresh LB plates and grown at 37°C with vigorous shaking overnight. To generate the biomass, two 2 L flasks containing 800 mL LB were inoculated to a starting OD_600_ of approximately 0.05 and bacteria were grown at 37°C with shaking. At the OD_600_ of 1.8–2.2 cells were collected by centrifugation at 10,000 *g* for 3 min (4°C, Beckman JLA-10.500 rotor), pellets were dissolved in 100 mL of ice-cold lysis buffer (10 mM Tris-acetate pH 8.0, 60 mM potassium glutamate, 14 mM magnesium acetate, 0.5 mM PMSF, 1mM DTT, pH 8.2), pelleted (3′ at 10,000 *g*, 4°C), taken up in 50 mL lysis buffer, and pelleted again in 50 mL Eppendorf centrifuge tubes (30′ at 3,000 *g* 4°C) yielding 4–5 g of biomass that was processed directly. To lyse the cells, lysis buffer was added to 4–5 g of cells to final volume of 12 mL, and cells were passed once though Stansted Fluid Power SFPH-10 Stansted Pressure Cell/Homogenizer at 2 bar. The lysate was clarified (10′ at 16,000 *g*, 4°C) and the supernatant was then desalted using Zeba Spin Desalting Columns 5 mL (ThermoFisher) equilibrated with lysis buffer. After adjusting A_260_ to 100 absorbance units with the lysis buffer, lysates were aliquoted in 50–100 μL fractions, snap frozen in liquid nitrogen and stored at −80°C.

### Polysome Profile Analysis

*B. subtilis* 168 and Δ*hpf* cell-free extracts were melted on ice and 6 AU_260_ units of each extract were loaded on 7–35% sucrose gradients made in HEPES:Polymix buffer (20 mM HEPES:KOH pH 7.5, 2 mM DTT, 5 mM MgOAc_2_, 95 mM KCl, 5 mM NH_4_Cl, 0.5 mM CaCl_2_, 8 mM putrescine, 1 mM spermidine; Antoun et al., 2004). Following centrifugation at 35,000 rpm for 2.5 h at 4°C (SW41 rotor, Beckman), the gradients were analyzed by measuring the continuous absorbance at 260 nm using a Piston Gradient Fractionator (Biocomp Instruments).

### Preparation of *S. cerevisiae* Cell-Free Extract

The lysate preparation protocol is based on that of Wu et al. (2007) with minor modifications. The wild type MBS and MJY1079 strains were grown in YPD medium at 30°C for 24 h. To generate the biomass, two 2L flasks containing 800 mL YPD were inoculated to a starting OD_600_ of approximately 0.001 and yeast were grown overnight at 30°C until OD_600_ of 4.0–7.0. Cells were collected by centrifugation (10′ at 7,000 *g*, 4°C), washed (3′ at 10,000 *g*, 4°C) three times with 50 mL of ice-cold lysis buffer (30 mM HEPES, 100 mM potassium acetate, 2 mM magnesium acetate, 0.5 mM PMSF, 1 mM DTT, pH 7.6), and pelleted in 50 mL Falcon centrifuge tubes (30′ at 3,000 *g*, 4°C) yielding 10 g of cells. Cells were frozen as small pellets by mixing with lysis buffer and dropped into liquid nitrogen and stored at −80°C or processed directly. To lyse the cells, 10 g of frozen cells were combined with 1 mL of frozen lysis buffer and crushed with a mortar and pestle in liquid nitrogen for 20 min. Note that more robust lysis methods have been shown to reduce the ability to translate exogenous mRNA in a yeast *in vitro* translation system (Leibowitz et al., 1991). The resulting lysate was transferred to a 50 mL falcon tube and incubated on ice until melted. The lysate was clarified (30′ at 3,000 *g* followed by ultracentrifugation of the supernatant for 10′ at 21,000 *g*, all at 4°C). The supernatant was desalted using Zeba Spin Desalting Columns 5 mL (ThermoFisher) equilibrated with lysis buffer. After adjusting A_260_ to 100 absorbance units with the lysis buffer, lysates were aliquoted in 50–100 μL fractions, snap frozen in liquid nitrogen and stored at −80°C.

### Preparation of *B. subtilis* Transcription-Translation System: Final Optimized Protocol

The protocol was based on that of Krinsky et al. (2016), with minor modifications. The translation system was assembled by mixing the *B. subtilis* lysate (see above) with the compound mix (10 mM Mg(OAc)_2_, 90 mM HEPES pH 8.2, 30 mM K_2_HPO_4_ pH 7.22, 135 mM potassium glutamate, 120 mM NH_4_OAc, 30 mg/mL PEG-8000, 1.8% (w/v) glucose, 3 mM DTT, 3 mM each of the 20 amino acid, 0.1 mM folinic acid, 3.5 mM ATP, 2.55 mM of GTP, CTP and UTP, 3 μg/mL plasmid pIVEX2.3MCs FFluc and 1.5 μM recombinant T7 RNAP) to a final volume of 30 μL per reaction point. The lysate and compound mix were combined at a 1:1 ratio. After gently mixing the binary system by pipetting, the reaction was incubated at 37°C for 1 h with shaking (500 rpm), and 10 μL of the reaction were added to 50 μL of Steady-Glo Luciferase Assay (Promega) (see below).

### Preparation of *S. cerevisiae* Translation System: Final Optimized Protocol

The protocol was based on that of Wu et al. (2007) with minor modifications. The translation system was assembled by mixing the yeast lysate (see above) with the compound mix (7 mM Mg(OAc)_2_, 20 mM HEPES pH 7.4, 200 mM KOAc, 2 mM DTT pH 7.4, 50 μM each of the 20 amino acids, 6 mM ATP, 0.5 mM GTP, 50 mM phosphocreatine, 40 μg/mL creatine phosphokinase, 10 μg/mL capped firefly mRNA with a 30 nucleotide poly(A) tail and 0.6 units/μL rRNasin) to a final volume of 30 μL per reaction. Prior to use, the lysates were treated with micrococcal nuclease (NEB) (24 units/μL) activated by 0.5 mM CaCl_2_ (final concentration) at room temperature in order to degrade cellular mRNA species. After a 20′ treatment with the nuclease, the reaction was stopped by addition of EGTA (pH 7.5) to a final concentration of 2 mM. Capped luciferase mRNA was refolded (7′ at 70°C and kept on ice prior to use) and added last to the compound mix immediately prior to assembling the final reaction mixture. The lysate and compound mix were combined to a 1:1 ratio. After gently mixing the binary system by pipetting, the reaction was incubated at 25°C for 1 h on an Eppendorf Thermomixer with shaking (500 rpm), and 10 μL of the reaction were added to 50 μL of Steady-Glo Luciferase Assay (Promega) (see below).

### Luciferase Assay

Steady-Glo Luciferase Assay (Promega) was used as per the manufacturer’s manual. The luciferase assay reagent was aliquoted in 50 μL fractions in 1.5 mL Eppendorf tubes kept in dark at room temperature. Readings were taken after addition of 10 μL reaction mixture to pre-aliquoted luciferase reagent using GloMax 20/20 Luminometer (Promega).

## Author Contributions

VM and VH conceived and coordinated the study. TB, VM, and VH drafted the manuscript together with input from MJ, TN, and HT. VM, TB, and VH designed the experiments and analyzed the data. TB, VM, MJ, and HT performed the experiments. All authors have read and approved the manuscript as submitted.

## Conflict of Interest Statement

The authors declare that the research was conducted in the absence of any commercial or financial relationships that could be construed as a potential conflict of interest.
